# Detection of Tumor Cell-Specific mRNA in the Peripheral Blood of Patients with Breast Cancer—Evaluation of Several Markers with Real-Time Reverse Transcription-PCR

**DOI:** 10.3390/ijms14011093

**Published:** 2013-01-08

**Authors:** Ulrich Andergassen, Simone Hofmann, Alexandra C. Kölbl, Christian Schindlbeck, Julia Neugebauer, Stefan Hutter, Verena Engelstädter, Matthias Ilmer, Klaus Friese, Udo Jeschke

**Affiliations:** 1 Klinik und Poliklinik für Frauenheilkunde und Geburtshilfe Ludwig-Maximilians-Universitaet Muenchen, Campus Innenstadt, Maistraße 11, 80337 Munich, Germany; E-Mails: ulrich.andergassen@med.uni-muenchen.de (U.A.); simone.hofmann@med.uni-muenchen.de (S.H.); alexandra.koelbl@med.uni-muenchen.de (A.C.K.); julia.neugebauer@med.uni-muenchen.de (J.N.); stefan.hutter@med.uni-muenchen.de (S.H.); verena.engelstaedter@uk-koeln.de (V.E.); klaus.friese@med.uni-muenchen.de (K.F.); 2 Frauenklinik, Klinikum Traunstein, Cuno-Niggl-Straße 3, 83278 Traunstein, Germany; E-Mail: christian.schindlbeck@klinikum-traunstein.de; 3 Department of Molecular Pathology, University of Texas MD Anderson Cancer Center, 7435 Fannin Street, Houston, TX 77054, USA; E-Mail: matthias.ilmer@googlemail.com

**Keywords:** breast cancer, circulating tumor cells, reverse transcription real-time PCR, marker genes

## Abstract

It is widely known that cells from epithelial tumors, e.g., breast cancer, detach from their primary tissue and enter blood circulation. We show that the presence of circulating tumor cells (CTCs) in samples of patients with primary and metastatic breast cancer can be detected with an array of selected tumor-marker-genes by reverse transcription real-time PCR. The focus of the presented work is on detecting differences in gene expression between healthy individuals and adjuvant and metastatic breast cancer patients, not an accurate quantification of these differences. Therefore, total RNA was isolated from blood samples of healthy donors and patients with primary or metastatic breast cancer after enrichment of mononuclear cells by density gradient centrifugation. After reverse transcription real-time PCR was carried out with a set of marker genes (BCSP, CK8, Her2, MGL, CK18, CK19). B2M and GAPDH were used as reference genes. Blood samples from patients with metastatic disease revealed increased cytokine gene levels in comparison to normal blood samples. Detection of a single gene was not sufficient to detect CTCs by reverse transcription real-time PCR. Markers used here were selected based on a recent study detecting cancer cells on different protein levels. The combination of such a marker array leads to higher and more specific discovery rates, predominantly in metastatic patients. Identification of CTCs by PCR methods may lead to better diagnosis and prognosis and could help to choose an adequate therapy.

## 1. Introduction

One major characteristic of malignant tissue is their differently regulated gene expression levels in comparison to normal tissue [1]. Such genes with altered expression have also been found in breast cancer with the help of microarray analysis screenings [2,3]. Among malignant diseases, breast cancer has the highest incidence worldwide and is the most frequent cause of death in women. However, the primary tumor is almost never lethal, whereas remote metastases and the total growing tumor mass lead to the patients’ death. Metastatic events occur when cells dissolve from the primary tumor, circulate via the blood stream or the lymphatic system to other organs, then evade into the new environment and become secondary tumors [4–6]. The incidence of these so-called “Circulating Tumor Cells” (CTCs) is linked to a worse prognosis for the patients’ survival time [7,8]. Thus, the detection of CTCs from peripheral blood samples could be a useful tool in diagnosis, prognosis and planning of further therapeutic steps. Since CTCs have largely the same genetic characteristics as the primary tumor and are therefore distinguishable from normal blood cells, a reverse transcription real-time PCR-based approach for the discovery of CTCs could constitute an easy, reliable and highly efficient method.

Here we present a TaqMan^®^ PCR assay using six marker genes which are known to be upregulated in breast cancer cells. We used Cytokeratin-8, -18 and -19 genes (CK8, CK18, CK19) known to be expressed on epithelial cells, such as CTCs, but not on blood cells. Moreover, immunocytochemical stainings (e.g., APAAP) use CK8, CK18, and CK19 routinely in cancer diagnosis [9,10]. As an additional marker, we used Mammaglobin (MGL) which is only expressed in the adult mammary gland and is known to be upregulated in breast cancer [11]. Synuclein gamma, also called Breast Cancer Specific Protein (BCSP), is highly expressed in advanced infiltrating breast cancer and is known as a marker for recurrence of the disease and formation of metastases [12–15]. BCSP-positive cells show a higher resistance to standard chemotherapy like paclitaxel than BCSP-negative/BCSP-low expressing cells [16,17]. The last marker used in this study is c-erbB2 (Her2), which is over-expressed in 20% of breast cancers and is also responsible for the aggressiveness of the tumor [18–20]. The big difference of Her2 expression in normal and cancer cells makes it a key target for several therapeutic approaches [21–23]. Furthermore Her2 was already shown to be a useful marker for Q-PCR, rendering equal or better values for detection sensitivity, specificity and positive and negative predictive values [24,25].

The above-mentioned markers were comparatively analyzed in blood samples withdrawn from adjuvant and metastatic breast cancer patients during surgery The gene expression levels of as well adjuvant as metastatic breast cancer patients were normalized to levels in blood samples of 20 healthy donors, considered as negative control group. Our intention was to detect differences in gene expression between the three sample groups and to find a signature of marker genes for CTCs in breast cancer by Real-Time-PCR.

## 2. Results and Discussion

### 2.1. Results

#### 2.1.1. Expression of CK8, 18, and 19 in Patients with Primary Carcinoma Undergoing Adjuvant Therapy

**Figure 1:**
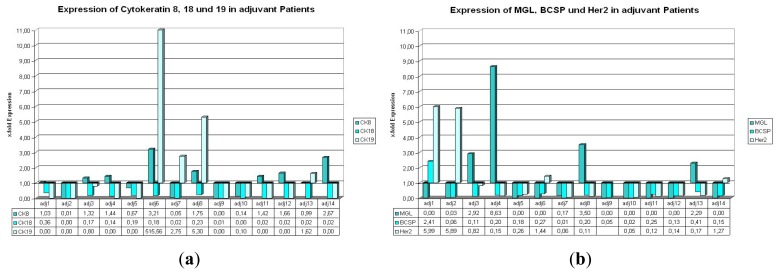
(**a**,**b**) Expression of the used marker genes in the adjuvant situation. (**a**) (**b**)

In the adj. group (respective tumor biomarker data are shown in Table 1), two samples show a simultaneous upregulation of CK8 and CK19 (Figure 1a, adj. 6 and adj. 8). In six further samples only CK8 and in two more samples only CK19 show relative expression values >1. In four cases all three examined genes are downregulated (Figure 1a, adj. 2, adj. 5, adj. 9 and adj. 10). Remarkably, all samples display expression values <1 or downregulation of CK18 in all patients. Furthermore, it is noteworthy that patient sample adj. 6 shows a more that 10-fold upregulated expression level of CK19.

#### 2.1.2. Expression of BCSP, Her2, and MGL in Patients with Primary Carcinoma Undergoing Adjuvant Therapy

**Figure 2:**
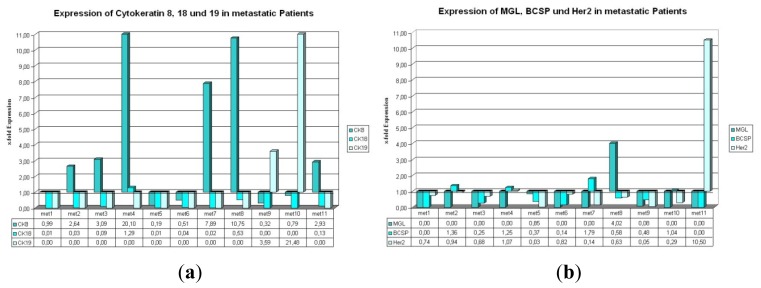
(**a**,**b**) Expression of the used marker genes in the metastatic setting. (**a**) (**b**)

In the same group of patients, we find only one case with two simultaneously upregulated genes (Figure 2, BCSP and Her2, adj. 1). In contrast to this, there are five cases with all three genes downregulated (Figure 1b, adj. 5, adj. 7, adj. 10, adj. 11, adj. 12). For one patient sample (adj. 9), no relative expression value could be calculated due to weak fluorescence signals that did not reach detection thresholds during the reverse transcription real-time PCR reaction. In only four cases MGL or Her2 show relative expression levels greater than 1 and in only one case (adj. 1) BCSP is upregulated in comparison to the reference level.

#### 2.1.3. Expression of CK8, 18, and 19 in Metastatic Patients

Regarding cytokeratin 8, 18, and 19 in metastatic patients (respective tumor biomarker data are shown in Table 2) CK8 is found upregulated in 6 out of 11 cases (Figure 2a, met. 2, met. 3, met. 4, met. 7, met.8, and met. 11); CK18 and CK19 show relative expression levels >1 in only one and two cases, respectively. In one sample (met. 4) CK8 and CK18 exhibit relative expression values suggesting an upregulation in comparison to the reference sample. For CK19 there is an intriguing finding: 9 out of 11 expression values were barely detectable and didn’t reach the threshold. In stark contrast to these findings, the other two expression values for CK19 (Figure 2a, met. 9 and met. 10) revealed a strong upregulation.

#### 2.1.4. Expression of BCSP, Her2, and MGL in Metastatic Patients

MGL, BCSP, and Her2 show a simultaneous downregulation in 5 out of 11 cases evaluated in this study. A concurrent upregulation of two genes can only be noticed in one case (Figure 2b, met. 4: BCSP and Her2). MGL alone shows an expression value >1 in only one case (met. 8), whereas 8 of the other 10 cases seem to have such low expression values, that further calculations were not possible, e.g., CK19 (Figure 2). BCSP and Her2 show higher expression levels than the reference sample in four and two cases, respectively (met. 2, met. 4, met. 7 and met. 10; met. 4 and met. 11).

### 2.2. Discussion

Real-Time PCR based techniques were already used for solid tumor profiling and are considered to be objective, robust and cost-effective molecular techniques, that could be used in daily cancer diagnostic routine [26]. We are now presenting a reverse transcription real-time PCR assay for the detection of Circulating Tumor Cells from peripheral blood, rendering the advantage for the patient, that no biopsies or bone marrow aspirations have to be withdrawn for the analysis.

Results of our study showed that cytokeratin genes seem to be the most promising markers for the detection of CTCs from peripheral blood of breast cancer patients with reverse transcription real-time PCR (TaqMan). The most suitable marker of the cytokeratin array used in our study is CK8 mRNA, rendering most expression values >1, whereas CK18 mRNA, in contrast, only revealed one significantly upregulated value in the metastatic group.

MGL, BCSP, and Her2 mRNA show few expression values >1 as well in adjuvant as in metastatic patients. However, in both settings we observed five samples where all three genes were simultaneously downregulated in comparison to the reference sample. Altogether, higher amplitudes for these three genes were detected in the adjuvant setting (Figure 1b*vs.*Figure 2b). CTCs can be detected from peripheral blood by Real-Time-PCR, using the cytokeratin markers, especially cytokeratin 8. But one single marker gene alone is not sufficient for PCR-based detection of circulating tumor cells from peripheral blood samples. Using a combination of different marker genes highly increases the chance for detection of CTCs, especially in samples of metastatic patients.

Some of the samples show different expressions for Her-2 in primary tumor *vs.* CTCs detected by Real-Time PCR (adj. 7–11, 13 and 14; met. 2–5, 7, 9 and 11; Figures 1b and 2b, Table 3). According to previous reports [27,28], a switch in Her-2 marker expression can occur. This phenomenon describes that CTCs can become positive for Her-2, even though the primary tumor is negative for Her-2 and vice versa. In the on hand study this switch can be seen in 50% of all samples in the adjuvant group and in 64% of the metastatic samples.

Another caveat of the present study is the small number of samples, which leads to a weaker statistical significance in the obtained results. A minimum number of 50 to 100 patients per group should be included in order to deduct statistically confirmed results. On the other hand, it is rather difficult to collect such a large number of blood samples, especially from metastatic patients.

To improve the detection of CTCs by Real-Time-PCR, more marker genes need to be tested; promising candidates are for example MMP13 [29–32], UBE2Q2 [33], Nectin-4 [34], and ALDH [35]. To avoid arduous collections of blood samples, both for physicians and patients, these markers should first be tested on blood samples mixed with different breast cancer cell line cells in a pilot *in vitro* study. Using these blood samples with a certain known number of cells derived from established breast cancer cell lines, standard curves could be collected and precious patient material could be saved at the same time. By the generation of standard curves a more accurate quantification of the gene expression values will also be possible. Here, we only focused on detection differences in gene expression levels between the sample groups, and found within this study two marker combinations for the adjuvant setting on and for metastatic patients. This method, already shown to be promising for multiparametric RNA analysis [36], could be of great clinical use for breast cancer diagnostics as well as prognosis and detection of micrometastasis using a multigene marker panel [37] and could serve as a further valuable tool to select appropriate and personalized therapeutic means.

Some more methods for CTC detection are described in the literature for example by immunomagentic cell separation [38], by EPISPOT assays [39], by Flow Cytometry followed by Real-Time PCR [40] and by immunomagnetic cell enrichment also followed by Real-Time PCR [41]. These methods, although they might have a higher sensitivity and specificity, are much more laborious, expensive, and time consuming. The advantage of the Real-Time PCR based method for CTC detection from peripheral blood presented in the on hand study, is furthermore, that by using different marker combinations, which should be tested as a next experimental step, not only detection but simultaneous characterization of the CTCs could be possible.

## 3. Experimental Section

### 3.1. Blood Samples

A total of 20 mL blood was withdrawn in EDTA-tubes from healthy donors (*n* = 20), patients with primary breast carcinoma undergoing adjuvant therapy (adj., *n* = 14), and metastatic patients (*n* = 11) Tumor Biomarker data are shown in Tables 1, 2 and 3. Blood samples were diluted to a volume of 50 mL with PBS (Biochrom, Berlin, Germany) and layered onto 2 × 25 mL Histopaque 1077 (Sigma-Aldrich, Taufkirchen, Germany), then centrifuged at 400× *g* for 30 min. Buffy Coat containing mononuclear cells was aspired and placed into a fresh tube. The suspension was filled up to 50 mL with PBS and spun down at 250× *g* for 10 min. This washing step was repeated once, then the supernatant was removed, the pellet was air-dried and frozen at −80 °C until use.

### 3.2. Ethics Approval

The study has been approved by the local ethics committee of the Ludwig-Maximilians University Munich (approval with the reference number 148-12) and has been carried out in compliance with the guidelines of the Helsinki Declaration of 1975. The study participants gave their written informed consent and samples and clinical information were anonymized for statistical workup.

### 3.3. RNA Isolation

Upon thawing, cell pellets were dissolved in 1 mL Trizol LS reagent (Invitrogen, Darmstadt, Germany) and 200 μL Chloroform (Merck, Darmstadt, Germany) was added. The suspension was mixed, stored on ice for 5 min and centrifuged at 12,000× *g* for 15 min at 4 °C. Upper clear phase was aspired and transferred into a fresh tube. A total of 500 μL cold isopropanol (Merck) and 2.5 μL glycogen (Invitrogen) were added. The solution was thoroughly mixed and frozen at −20 °C over night. RNA was precipitated by centrifugation at 12,000× *g* for 10 min at 4 °C. After removal of supernatant RNA-pellets were washed with 1 mL 75% Ethanol (Merck) by centrifugation at 12,000× *g* for 8 min at 4 °C. Then pellets were air-dried and dissolved in 20 μL DEPC-treated water. Concentration and ratio of isolated RNA were measured by Nanodrop-System-Nanophotometer (Implen, Munich, Germany), inhibition controls (by sample dilution) were carried out and RNA integrity was controlled by denaturing formaldehyde-gel electrophoresis.

### 3.4. Reverse Transcription

For reverse transcription an RNA-amount of 5 μg in a maximum volume of 8 μL in DEPC treated water was used. A total of 10 μL 2× RT reaction mix (containing oligo(dT)_20_ (2.5 μM), random hexameres (2.5 ng/μL), 10mM MgCl2 and dNTPs) and 2 μL RT enzyme mix (including SuperScript™ III RT and RNaseOUT™) (SuperScript III First Strand Synthesis Super Mix; Invitrogen) were added. The solution was incubated at 25 °C for 10 min and at 42 °C for 50 min. Following this step, polymerase was heat-inactivated at 85 °C for 5 min and subsequently chilled on ice. A total of 1 μL (2U) RNase H was added to the reaction and the whole solution was incubated at 37 °C for 20 min. The obtained cDNA was either subsequently processed or stored at −20 °C until use.

### 3.5. Nested PCR

We performed a nested PCR for marker CK 8, 19 and BCSP. A total of 1μl of the prepared cDNA was mixed with 1 μL primer, 10.5 μL nuclease free water and 12.5 μL 2× PCR mastermix (Promega, Madison, WI, USA). The samples were processed on Eppendorf Master Cycler (Eppendorf, Hamburg, Germany). Settings for the PCR runs are: one enzyme activation cycle at 98 °C for 30 s, 34 cycles at 98 °C for 15 s, 57 °C for 30 s, 72 °C for 2 min and one terminatory cycle at 72 °C for 5 min. The obtained PCR product was either processed or stored at −20 °C until use. The primer sequences are presented in Table 4.

### 3.6. Real-Time RT-PCR (TaqMan^®^)

Real-Time RT-PCR reactions were carried out as quadruplicates in a Fast Optical 96-well plate (Applied Biosystems, Foster City, CA, USA). A total of 1 μL of the prepared cDNA was mixed with 1 μL TaqMan^®^ Gene Expression Assay (CK18 (Hs_01920599_gH), Her2 (Hs_00170433_m1) MGL (Hs_00419570_m1) and GAPDH (Hs_99999905_m1)), 10 μL TaqMan^®^ Fast Universal PCR Master Mix (all Applied Biosystems), and 8 μL PCR-grade water per sample was prepared and pipetted into the respective wells on a PCR plate. The same mix was used for the housekeeping/reference gene B2M [42,43] together with a FAM-Tamra primer mix (Biomers, Ulm, Germany). For CK8, 19 and BCSP the PCR product of the nested PCR were inserted for real-time RT-PCR. Primer sequences are presented in Table 5. After its sealing with an adhesive cover (Applied Biosystems), samples were analysed on a 7500 Fast Real-Time PCR machine (Applied Biosystems). We used an established setting for the PCR runs: One enzyme activation cycle at 95 °C for 20 s, 40 cycles at 95 °C for 3 s and 60 °C for 30 s. Fluorescence was measured in every step and displayed by the provided SDS 1.3.1 software (Applied Biosystems, 2001–2005, Foster City, CA, USA). Non-template-, and-RT controls were also implemented in the experimental setting. PCR efficiency of TaqMan Primers from Applied Biosystems is stated with 100% ± 10% by the provider. PCR efficiency of RealTime RT-PCR Primers for CK8, CK19, BCSP and B2M was analyzed by a standard curve assay and is shown in Table 6.

### 3.7. Evaluation

Data from SDS software V.1.3.1 were transferred into Microsoft® Excel™, evaluated, and clustered column charts generated. Relative expression rates are calculated by 2^−Δ ΔCT^ formula [44]. Relative expression values >1 were considered upregulated for the respective gene in a certain sample in comparison to negative control samples. Negative controls consisted of mean gene expressions of blood samples spent by healthy donors. Non-template control and RT control assays did not yield any fluorescent signals.

## 4. Conclusions

In the future more marker genes will have to be tested for their benefit in the detection of breast cancer cells in the blood stream. A combination of various markers could potentially help detect tumor-cell-specific mRNA, as already described by Gervasoni *et al.* [45] and Bölke *et al.* [46]. Taking these considerations into account, the method presented in this study contributes largely to detection of cancer cells in patients’ blood. Ultimately, detailed characterization of CTCs leads to better diagnosis as well as prognosis and could help in choosing the adequate therapy.

## Figures and Tables

**Table 1:** Tumor Biomarker Data of adjuvant patients.

Patient	Histology	T-stage	N-stage	Her2-status	Estrogen receptor (%)	Progesterone receptor (%)
Adj.1	Inv. ductal	pT2	pN3	+++	0	0
Adj.2	Inv. ductal	pT1c	pN0	++	90	90
Adj.3	Inv. ductal	pT2	pN0	-	90	90
Adj.4	Inv. lobular	pT1c	pN0	-	95	90
Adj.5	Inv. ductal	pT2	pN0	-	30	50
Adj.6	Adeno-squamous	pT3	pN0	+++	0	0
Adj.7	Inv. ductal	pT1c	pN3a	+	80	50
Adj.8	Inv. ductal	pT2	pN3a	+++	30	10
Adj.9	Inv. ductal	pT2	pN0	+	0	10
Adj.10	Inv. ductal	pT1c	pN0	++	90	60
Adj.11	Adenocarcinoma Lobular	pT2	pN1	+	80	30
Adj.12	Inv. ductal	pT2	pN0	-	90	30
Adj.13	Inv. ductal	pT2	pN0	+	80	80
Adj.14	Inv. ductal pT1c	pN0	-	0	0	

**Table 2:** Tumor Biomarker Data of metastatic patients.

Patient	Histology	T-stage	N-stage	Metastases	Her2- status	Estrogen receptor (%)	Progesterone receptor (%)
Met.1	Inv. ductal	pT2	pN0	Liver, Bones	-	0	0
Met.2	Inv. ductal	pT2	pN0	Brain	+++	0	0
Met.3	Inv. ductal	pT4	pN0	Bones	+	0	0
Met.4	Inv. ductal	pT4	pN1	Lung	++	0	0
Met.5	Inv. ductal	pT4	pN0	Lung	+	60	40
Met.6	Inv. ductal	pT1c	pN0	Bones, Liver	-	0	0
Met.7	Inv. ductal	pT2	pN0	Liver	+	0	0
Met.8	Inv. ductal	pT3c	pN1	Liver, Bones, Brain	-	0	0
Met.9	Inv. ductal	pT3	pN0	Bones	+++	20	10
Met.10	Inv. ductal	pT2	pN1	Lung, Bones	-	30	10
Met.11	Inv. ductal	pT1b	pN0	Bones	-	90	70

**Table 3:** Number and percentages of c-erbB2 (Her2)-switches between primary tumor and circulating tumor cells (CTCs).

Her2 Status Tumor/CTCs	Adjuvant		Metastatic	
	
	Number	%	Number	%
+/+	3/14	22%	1/11	9%
+/−	1/14	7%	6/11	55%
−/+	6/14	43%	1/11	9%
−/−	4/14	28%	3/11	27%

**Table 4:** Primer used for nested PCR.

Gene	Forward primer	Reverse primer
CK8	5′-cgtcaagctgctggac-3′	5′-aggctgtagcggccgg-3′
CK19	5′-gcctggttcaagccgg-3′	5′-ctcctgattcccgctc-3′
BCSP	5′-acactgtgtggccaagac-3′	5′-ccactctgggtctgcc-3′

**Table 5:** Primer & Probes used for Real-Time RT-PCR.

Gene	Forward primer	Reverse primer	Hydrolysis probe
B2M	5′-ggccgagatggctccg-3′	5′-gatgaaaccccatagc-3′	5′-aggctatccagattca-3′
CK8	5′-cctacaggaagctgga-3′	5′-gctcagaccaatagcc-3′	5′-ggagagccggggagtc-3′
CK19	5′-ccgaggttacacctgc-3′	5′-gatcagcgccgatatg-3′	5′-ctgagcatgagctgcc-3′
BCSP	5′-ggagaacatcgtcacc-3′	5′-ggatgcctcactcctg-3′	5′-tgcgcaaggagaggcc-3′

**Table 6:** PCR efficiency of Real-Time RT-PCR Primers drawn by standard curve assay.

Gene	*R*^2^-value	PCR efficiency
B2M	0,9845	98,45%
CK8	0,9923	99,23%
CK19	0,9066	90,66%
BCSP	0,9894	98,94%
